# Social/dialogical roles of social robots in supporting children’s learning of language and literacy—A review and analysis of innovative roles

**DOI:** 10.3389/frobt.2022.971749

**Published:** 2022-10-05

**Authors:** Katharina J. Rohlfing, Nicole Altvater-Mackensen, Nathan Caruana, Rianne van den Berghe, Barbara Bruno, Nils F. Tolksdorf, Adriana Hanulíková

**Affiliations:** ^1^ Developmental Psycholinguistics, Faculty of Arts and Humanities, Paderborn University, Paderborn, Germany; ^2^ Developmental Psychology, Psychologisches Institut, Johannes-Gutenberg-Universität Mainz, English Linguistics, University of Mannheim, Mainz, Germany; ^3^ School of Psychological Science, Macquarie University Centre for Reading, Macquarie University, Sydney, NSW, Australia; ^4^ Urban Care & Education, Windesheim University of Applied Sciences, Almere, Netherlands; ^5^ CHILI Lab, EPFL, Lausanne, Switzerland; ^6^ Language and Cognition, Deutsches Seminar, Albert-Ludwigs-Universität Freiburg, Freiburg, Germany

**Keywords:** dialogical roles, human–robot social relationship, child–robot interaction, language learning, literacy, social roles

## Abstract

One of the many purposes for which social robots are designed is education, and there have been many attempts to systematize their potential in this field. What these attempts have in common is the recognition that learning can be supported in a variety of ways because a learner can be engaged in different activities that foster learning. Up to now, three roles have been proposed when designing these activities for robots: as a teacher or tutor, a learning peer, or a novice. Current research proposes that deciding in favor of one role over another depends on the content or preferred pedagogical form. However, the design of activities changes not only the content of learning, but also the nature of a human–robot social relationship. This is particularly important in language acquisition, which has been recognized as a social endeavor. The following review aims to specify the differences in human–robot social relationships when children learn language through interacting with a social robot. After proposing categories for comparing these different relationships, we review established and more specific, innovative roles that a robot can play in language-learning scenarios. This follows Mead’s (1946) theoretical approach proposing that social roles are performed in interactive acts. These acts are crucial for learning, because not only can they shape the social environment of learning but also engage the learner to different degrees. We specify the degree of engagement by referring to Chi’s (2009) progression of learning activities that range from active, constructive, toward interactive with the latter fostering deeper learning. Taken together, this approach enables us to compare and evaluate different human–robot social relationships that arise when applying a robot in a particular social role.

## 1 Introduction

A large body of research points to the success of robots designed for the purpose of education (Mubin et al., 2013; Belpaeme et al., 2018; Kanero et al., 2018; Lee and Lee, 2022). Existing reviews have identified a variety of application domains going hand in hand with different roles for the robots. For learning, Mubin and colleagues (2013) characterize these roles as levels of involvement of the robot, and these authors differentiate between a passive robot that can be used as a tool to be programed and a “co-learner” (*p*. 3) that is active, can be more involved, and can influence the learning process. Ahmad and colleagues (2021) summarize the social roles that a robot can fulfill in learning as being a teacher or tutor, a learning peer, or a novice (see Section 2 for more details on established roles). Roles determine the robot’s behaviors, but also its responsibility for, and thus contribution to, the learning process. To fulfill the role of a teacher, for example, a robot has to initiate the interaction and guide the learner toward becoming knowledgeable on the taught content. Ahmad et al. (2021) further propose that the decision for one role over another depends “on the content, the tutor or instructor, the form of student and the essence of the learning process” (*p*. 295). Hence, the design of the role that a robot fulfills in a learning process clearly has manifold consequences not only for the robot’s appearance but also for the interaction and the learning process. Despite these far-reaching consequences, and even though the literature offers different forms of designing an interaction with a robot for the purpose of enjoyable (Lin et al., 2022) and successful learning, little is known about whether and how these different roles can be designed systematically, let alone how they differ in shaping a human–robot social relationship (De Graaf, 2016; Tolksdorf et al., 2020).

In the following review, we aim to specify the differences between the roles for a robot by proposing categories that make it possible to perform a systematic comparison. We thereby follow Mead’s (1946) theoretical account according to which any interaction brings about a role. This role is both social and dialogical. It is social, because it reflects a relation of an individual to a social group (Mead, 1946, *p*. 164). For example, by being a tutor, a person has to teach a learner and is considered to be more knowledgeable than the learner. This relation shapes attitudes and expectations. These become observable in interactive acts in the form of verbal and nonverbal communicative behaviors directed toward the others. This is why a social role is also dialogical. In other words, whereas attitudes and expectations are socially motivated, they are accessible through communication. When interacting with others, the performance of interactive acts is influenced by the role. For example, a tutor will provide an explanation, whereas a learner can ask questions.

Extending what is known about the roles and the way they shape interactive acts, this review aims to differentiate the abilities that serve these roles. This extension contributes a framework for the design of social robots that should raise awareness among scientists, developers, and users as to what kind of capabilities need to be implemented for what kind of interaction to serve what kind of educational purpose. Accordingly, in Table 1, we differentiate the “perceptual”, “cognitive,” and “dialogical” abilities that need to be implemented in a robot in order to fulfill a particular role: Whereas “perceptual” abilities enable a robot to perceive specific communicative signals, “cognitive” abilities can be implemented in a variety of ways leading to different levels and complexities in processing the perceived information. Finally, with “dialogical” abilities, robots are able to engage with a social partner.

**TABLE 1:** Definition of innovative roles and references to the existing literature.

Role	Definition of the role	Required abilities of the robot to fulfill the role			Studies on language learning and literacy
		Perceptual	Cognitive	Dialogical	
Socioemotionally supporting	Robot offers socioemotional support to alleviate anxiety and promote engagement during learning	To perceive, at a minimum, whether the child is engaged in the learning activity. This will allow the robot to respond/interact appropriately at key points in the activity, or to identify when additional encouragement is needed if the child has become distracted/disengaged. It would be additionally beneficial, but not essential, for robots to perceive mood states in children	To be capable of modeling the optimal frequency of feedback and animacy during a learning activity. This will enable robots to provide enough feedback to facilitate engagement without the risk of this form of feedback or animation form becoming a distraction. This is important, because robots serving this role are not directly supporting the learning processes and so should not be the child’s primary focus of attention	To initiate dialogue that can demonstrate the robots’ sentience and own engagement in the learning activity (to promote task engagement and trust in robot competence). Dialogue could also offer encouragement/reassurance when child’s engagement in task stalls. Verbal adaptation of robot speech (e.g., volume, pitch) can also improve rapport, the social context, comfort, and learning	Caruana et al. (2022); Chen et al. (2020)
Assisting	Robot offers socioemotional assistance to learner (learner assistant) or takes over tasks from teacher (teaching assistant)	To perceive and record learner behavior	To interpret and assess behavior in terms of task performance; to provide appropriate input	To initiate and/or maintain dialogue; to offer encouragement and/or help; to provide feedback	Alemi et al. (2015); Deublein et al. (2018); Engwall and Lopes (2022); Hong et al. (2016); Hsiao et al. (2015)
Prompting	Robot invites the learner to use language expressively	To understand what the learner is saying. It would be additionally beneficial if it could detect pronunciation errors in the learner’s speech and provide feedback	To interpret learners’ speech; to provide appropriate input; to explicitly invite the learner to speak	To initiate and/or maintain dialogue; to offer encouragement and/or help; to provide feedback on communicative skills in general or on pronunciation	Lin et al. (2022)
Role playing	Robot acts out a certain role by collaboratively negotiating the plot and meaning	To recognize nonverbal social signals such as facial expressions, gestures, posture, pointing, eye movements	To be trained to produce proactive and socially appropriate behavior	To respond adequately and to predict what type of interaction and behavior it should evoke from a child in a given role play, it should provide hints and encouragement in a specific role	Ali et al. (2019); Lee et al. (2011)
Displaying incorrect behavior	Robot acts as an error-prone tutee for the human tutor	To understand the instructions/explanations/corrections given by the human tutor verbally, via gestures, and/or via a mediation tool	To improve on its performance proportionally and according to the received guidance, without ever surpassing the performance of the human tutor	To establish a cooperation cycle with the human tutor to converge on a performance deemed satisfactory by the tutor	Tanaka and Matsuzoe (2012)
Encouraging metatalk	Robot initiates an interaction/dialogue in which communication either becomes the focus of the communicative activity, is manipulated, or is reflected upon	To perceive the child’s utterances or to deliberately initiate certain peculiarities (e.g., longer response times) in its perception that can be reflected upon	To model child’s actions and infers knowledge related to task; to suggests appropriate lessons for demonstration	To initiate/elicit a specific type of dialogue and engage in an exchange about the communicative situation	Horwath et al. (2018); Hsiao et al. (2015); Ramachandran et al. (2018); Spaulding et al. (2021)

In addition to our claim that roles shape an interaction, we consider learning processes to require a particular awareness of roles. This is because the interactive acts performed are crucial: Not only can they shape the social environment of learning, but they also engage the learner to a different degree (Chi, 2009). The degree of engaging the learner can be specified more clearly when following Chi (2009), who proposed three forms of engaging a learner in the construction of knowledge. Through analyzing the acts performed by tutors and learners in detail and across many studies, Chi (2009, *p*. 73) formulated a conceptual framework for differentiating learning activities in terms of observable “overt activities and underlying learning processes.” Within this framework, activities can be differentiated into active (doing some physical movements while learning), constructive (producing additional output with contents that go beyond the given information), and interactive (participating in a dialogue characterized by exchange and co-construction of follow-up activities such as defending a position, elaborating, etc.). These overt activities differ with respect to the underlying learning processes (Chi, 2009, *p*. 77): In active activities, attending processes are elicited. Their function is to activate existing knowledge or to store new information. In constructive activities, new knowledge inferences or integrations are elicited. With these kinds of activities, it is necessary to organize one’s own knowledge in order to gain coherence. Finally, interactive activities elicit “creating processes” (*p*. 77) in which it is necessary to incorporate a partner’s contribution. Based on studies comparing the different forms of activities, Chi (2009) suggests that for deeper learning to occur, interactive activities are required. To the best of our knowledge, activities initiated by social robots have barely been analyzed with respect to what cognitive processes they elicit and how they might thus foster learning. In Table 2, we describe different forms of activities that can guide the design of a robot depending on what kind of interaction (and thus other forms of learning) is intended. Following Mead’s (1946) suggestion that a particular role should be seen as reflecting one’s position within a group, we critically reflect on how these activities change the social context and might shape the role of the other group members involved. For example, when a robot can provide an individual treatment for a child’s limited vocabulary, a teacher or educator in kindergarten might feel less responsible for fostering this area of language. Another example could be that the presence of a social robot fulfilling a particular role opens up new possibilities for how other children can be involved.

**TABLE 2:** Potential of innovative roles with respect to engagement and involvement of others.

Role	Learning/Engagement			Shaping the social context: Involvement of others
	Active	Constructive	Interactive	
Socioemotionally supporting	Periodic prompts, generic feedback, or progress updates (e.g., duration or proportion of task completed)	Prompting children to reflect on learning success/achievements at appropriate points during the learning activity	Sharing the learning activity, including asking clarification questions, making errors to elicit feedback, and establishing a social context that is nonjudgmental	Likely to be most effective if able to deploy autonomously safely and effectively, without the need for an adult to control it. This is because the key benefit of support robots is that they can offer a social agent that can co-experience the activity but is less likely to be perceived as judgmental/intimidating
Assisting	Providing or highlighting input	Prompting learner to act on material or to engage in task	Providing feedback, co-solving a task	Shift the role of the teacher from instructor to moderator of learning
Prompting	Periodic prompts, generating questions or topic suggestions	Engaging the learner in problem-solving discussions (e.g., riddles); prompts should be fruitful in new contexts	Engaging in discussions (e.g., developing a stance toward a subject)	Likely to be most effective if learners have some previous language knowledge, to engage in more elaborate conversations with the robot. Could work both one-on-one and in small groups
Role playing	Generating questions, topic suggestions, asking for help	Constructing common ground	Negotiating and altering meaning, exchanging roles	Possible involvement of other children to simultaneously engage in peer interactions as well as robot–child interactions. Adults might be necessary in case of younger children
Displaying incorrect behavior	Asking for feedback and guidance in the task	Engaging the learner in reflection and explanations	Sharing the learning activity including asking clarification questions, making errors to elicit feedback, and establishing a social context that is nonjudgmental	Likely to be most effective if able to deploy autonomously, safely, and effectively, without the need for an adult to control. Possible involvement of other learners in collaborative tutoring of the robot to allow for engaging peer interactions alongside the robot–child interactions
Encouraging metatalk	Providing or highlighting some kind of linguistic context such as a narrative, word, sentence structures, or communicative practices that can be talked about	Engaging a child in reflection and discussion about the linguistic context (with a peer)	Providing feedback and co-constructing new knowledge by scaffolding the child; e.g., asking the child to explain a subject to the robot	Possible involvement of a caregiver of the child by discussing/reflecting on the linguistic utterances of the robot or the interaction/turn taking with the robot

In the following, we first review the most common roles that a social robot fulfills in current research (Section 2). Adding to the existing roles, we further review innovative roles that a robot can fulfill for the purpose of promoting language learning and literacy (Section 3). First, the selection of the innovative roles is driven by the intention to extend the already well-established roles of a robot as a learner, tutor, or peer in RALL (robots assisting language learning). We opted for roles that can be specified in overt dialogical acts. Instead of characterizing a robot as a tutor, we propose acts with which a robot can assist a tutor or a teacher—that is, assisting (Section 3.1) and supporting (Section 3.2). Tackling on novel aspects of assistance and support, we review possibilities of assisting also children. With regard to supporting activities, the review focuses on socioemotional aspects that have barely been considered in the design of robots so far. Second, our selection of the roles for the review was motivated by aspects from developmental studies. These aspects are reflected in four roles that strengthen the child’s own engagement and learning strategies: prompting (Section 3.3) that follows caregivers’ intuitive behaviors, role playing (Section 3.4), purposefully making mistakes (Section 3.5), and encouraging metatalk (Section 3.6). The order of the presented roles can be seen as a progression from tutor- to child-oriented, thus, increasingly empowering children in their participation that is crucial for learning (Chi, 2009).

We describe and review the established and innovative roles by first specifying different requirements imposed on robots’ perceptive, cognitive, and dialogical abilities. We argue that the established roles are too coarse-grained to further specify all the abilities needed. In contrast, the abilities can be further specified for the innovative roles (Table 1 provides a summary). Furthermore, we analyze the potential of the innovative roles to evoke interactive acts (summarized in Table 2) that we evaluate according to a taxonomy proposed by Chi (2009). Finally (also in Table 2), we analyze what impact the innovative roles have on the social environment and how they make an engagement of others possible or necessary. Using this review, researchers in robotics and developers of social robots can compare and evaluate different dialogical roles in order to have a better basis for a decision on the robot’s desired educational effect in language and literacy learning.

## 2 Established dialogical roles that a social robot fulfills

### 2.1 Social robot as tutor

Certainly, the most common role that a robot can fulfill in a child–robot interaction for language learning and literacy is to serve as a knowledge resource (e.g., Movellan et al., 2009; Vogt et al., 2017; 2019 in the first learning session). A meta-analysis by Belpaeme and colleagues (2018) revealed that in 86% of studies on robots for education, the robot was designed to serve the role of a tutor or a teacher. Accordingly, a robot acts as a tutor and a more knowledgeable partner “to foster the acquisition of new knowledge and skills” (Chen et al., 2020, *p*. 3). Chen et al. (2020) rightly point out that the learner gains from an interaction with a skilled tutor because of the guidance and scaffolding provided in the learning process. For this, tutors need to fine-tune to the learner’s skills in a way that is “temporarily assisting learners to achieve new skills or levels of understanding they would not reach on their own” (Schodde et al., 2019, *p*. 1). First approaches to a tutoring robot that can scaffold the learner’s behavior are being developed (Schodde et al., 2019; Cumbal, 2022). They require highly nested abilities from a robot, and their depiction goes beyond the categories proposed in Table 1. More specifically, they require not only a model of the learner’s perceptual, cognitive, and linguistic skills but also a model of the task. For scaffolding, both models do not just need to be combined. Verbal guidance also needs to be derived from the partner model and designed in a way that includes nonverbal behaviors and can adjust to children’s linguistic capabilities (Norman et al., 2021; Rohlfing et al., 2021).

Although existing studies speak to the high potential of robots for teaching language and literacy, Kanero et al., 2018 summarize critical points regarding this research: One is the lack of evidence (or control) that robots are more effective in the long term than other options. A further critical point relates to the exposure of children to technology rather than to human relationships (Sharkey, 2016). In this respect, Sharkey (2016, *p*. 295) concludes that there are good reasons not to encourage fully fledged robot teachers.

### 2.2 Social robot as peer

A group of scholars (Kory Westlund et al., 2016; Belpaeme et al., 2018) suggest that the decision for one role over another should be based on whether the interaction with the robot is perceived as being fun while, at the same time, effective to achieve the learning goal. The suggested “framing” (Belpaeme et al., 2018, *p*. 331) corresponds to research in developmental studies revealing that children tend to treat a robot as a peer and social actor (Tanaka and Matsuzoe, 2012; Breazeal et al., 2016; Oranc and Küntay, 2020). This preference is likely to lead to acceptability of a robot (Belpaeme et al., 2018) but bears some limitations, because it is especially pronounced in young children (Shin and Kim, 2007).

The role of a peer is motivated by its higher acceptability in children, and it is often contrasted with that of a tutor to emphasize the companionship (Admoni and Scassellati, 2014). According to Belpaeme et al.’s (2018) meta-analysis, robots were designed to fulfill the role of a peer or a novice only in 9% of the studies investigating robots for educational purposes. The characteristic of a peer interaction is that both partners are responsible for knowledge construction (Chi, 2009). Thus, they both need to be engaged. The motivation for the first robot fulfilling the role of a peer was to establish a relationship between the robot and the children (Kanda et al., 2004). The authors considered the robot’s ability to identify and recognize its peers as a prerequisite for this relationship that could only evolve over time. The ability to identify and recognize is based on perceptual and cognitive skills. For dialogic skills, Baxter and colleagues (2017) found that personalized robots are a better design to engage children in a learning interaction. With “personalization,” the authors refer to “adaptation of non-verbal behavior, personable language content, and alignment to task performance” (Baxter et al., 2017, *p*. 4). Clearly, the design of a robot as a peer demands all abilities to be responsive to the partner in order to achieve cooperation. However, in current research, beyond its emphasis on engagement, it is not exactly specified what kind of activities the role should bring about to foster what kind of learning process. In Section 3, we therefore suggest that the coarse-grained demands on cooperation can be broken down into some specifics.

### 2.3 Social robot as learner

What is established in current research is that a robot can fulfill the role of a less knowledgeable partner, and a learner. A meta-analysis of robots in education has suggested that a robot in this role can support skill consolidation and mastery (Belpaeme et al., 2018). In fact, some studies have investigated whether particular roles of a robot—as much enjoyment and engagement as they may provide—are beneficial for learning. Tanaka and Matsuzoe (2012), for example, put children into a teaching role and investigated learning-by-teaching effects when Japanese children taught some English words to a “care-receiving robot” (*p*. 78). To leverage the child’s knowledge to the level of an expert in an interaction is clearly engaging but might also foster empathy and compassion (Lee et al., 2019).^1^

Tanaka and Matsuzoe (2012) found that a robot fulfilling the learner’s role can foster English verb learning in Japanese children. Whereas these studies add a lot to the growing possibilities of applying a robot, they lack a clear comparison to other potential roles. In addition, for future research, it remains an open question whether the learning effect is relying on the teaching and caring activity that requires a tutor to adapt to the learner or on the knowledge construction ability of the learner that is feedbacked to the tutor. For the role of an active learner, it is necessary to display an increase of learning by, for example, responses that are “substantive and meaningful” (Chi, 2009, *p*. 82). The implementation of such responses reflecting cognitive and/or dialogical processing is lacking in the current design of robotic learners.

**FIGURE 1:**
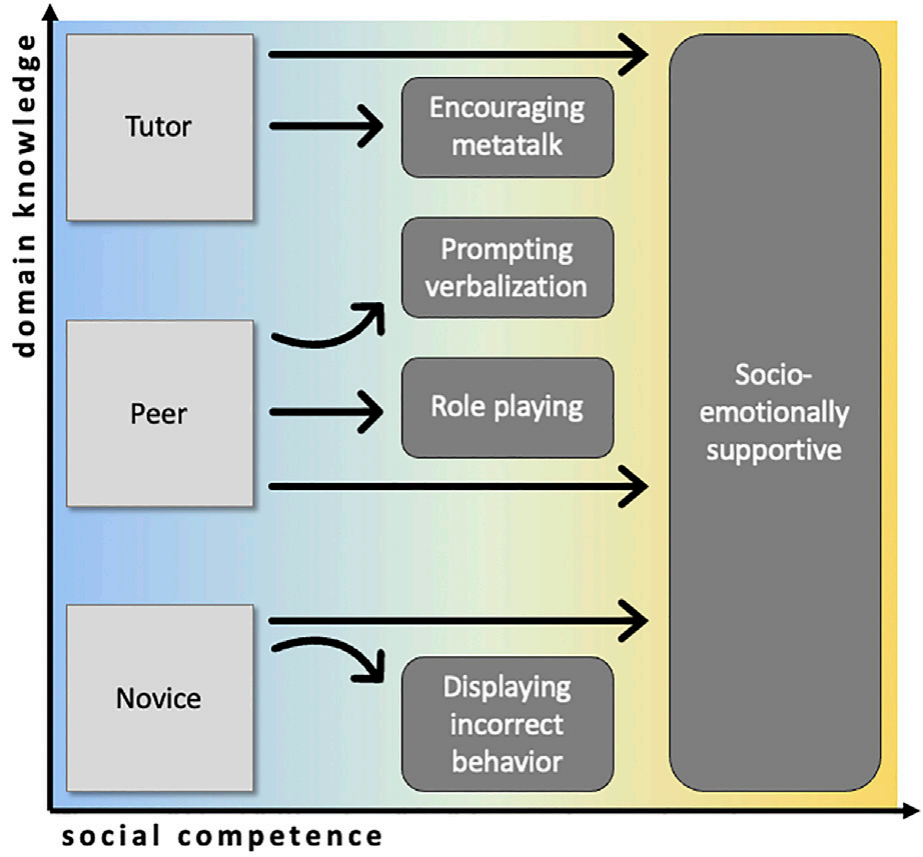
Summary of the various social/dialogical roles presented in this review. On the left, the established roles differ with respect to the domain knowledge. They can be further specified in the innovative roles that differ in terms of social competence.

To summarize, the established roles offer a coarse-grained differentiation into three roles (see Figure 1). However, as highlighted in Section 2.1, these roles demand nested skills that currently cannot be implemented in a satisfactory manner in a robot. With the innovative dialogical roles in Section 3, we propose an extension to the established roles. They make some aspects of the roles feasible and specify the required capabilities in Table 1. At the same time, they are innovative, because they uncover abilities that further extend the established categories (see Figure 1) toward the social competence (on a horizontal axis), thereby bringing in more fluid domain knowledge (on a vertical axis). Thus, they offer novel forms of education for the purpose of language learning and literacy, even though the roles proposed below do not exhaust the possibilities that the two dimensions in Figure 1 can yield. By focusing on what abilities foster what kind of activities that can be applied in learning interaction, we add to the current literature that offers little discussion on whether and how the different roles can be designed systematically.

## 3 Innovative dialogical roles that a social robot can fulfill

### 3.1 Using a social robot to assist learning

A role that seems implicit in most applications of social robots is that of assistant in service (Čaić et al., 2019), care (Broekens et al., 2009), or education (Belpaeme et al., 2018). In the role of an assistant supporting language learning and literacy development, robots can be a tutor, novice or peer (Van den Berghe et al., 2019; Neumann, 2020). Yet, the specific potential or contribution of the assistant role per se has rarely been addressed in detail. In educational settings, assistance can be considered in (at least) two ways: focusing either on assisting the *teacher* to support instruction or on assisting the *learner* to solve a given task. Both of these roles arguably entail unique opportunities and challenges, and we will elaborate on them below.

Considering the *teacher-assisting* role, the robot often acts as a tutor providing learning material in a classroom setting allowing the teacher to focus on student performance rather than on instruction (Alemi et al., 2015; Hong et al., 2016). Hence, the robot assists the teacher by taking over specific instructional tasks, thereby freeing educational resources. In classroom settings, the robot could further collect, process, and monitor data on learning performance and progress that might inform subsequent teaching on either the group or individual level. Considering such personalization, robots have the potential to scaffold learning by providing learning content that is tailored to individual abilities (Gordon and Breazeal, 2015). The use of social robots in interventions such as the treatment of dysgraphia (Gargot et al., 2021) can be understood in a similar vein. Further functions that teaching assistant robots may take over include the role of an invigilator who supervises student actions (Ahmad et al., 2021), as a native speaker in second-language learning that models language behavior and elicits language production (Han et al., 2012; Spaulding et al., 2018), or as catalyst that facilitates the interaction between learners in a setting in which the teacher is not actively involved (Engwall and Lopes, 2022). In all these settings, the robot may be perceived as less intimidating and judgmental from the learners’ perspective as well as more motivating and engaging than the human teacher (see Sections 2.2 and 3.2). Importantly, because the robot might reduce the educational and cognitive load of teachers, their assistance makes it possible to shift the role of the teacher from instructor to moderator of learning. In Chi’s (2009) taxonomy, the teaching assistant might, thus, allow the teacher to build more constructive and interactive learning contexts.

Considering the *learner-assisting* role, the robot can provide direct support when the learner encounters difficulties by providing information to help solve the problem at hand (Crossman et al., 2018). For instance, the robot can use locational or verbal cues to focus the learner’s attention on relevant material in the input (Hemminghaus and Koop, 2017). Robots might also support learning indirectly by giving feedback on task performance (Gordon and Breazeal, 2015; Hsiao et al., 2015), by mirroring learner behavior or providing a different perspective (Wood et al., 2017), by increasing motivation and engagement (Deublein et al., 2018), or by focusing and redirecting attention to the task when the learner drifts off. In an embodied way, a robot might further compensate for impairments by, for example, reading out text for a visually impaired learner and representing or connecting a learner who cannot be physically present in a class (Newhart et al., 2016). In all these scenarios, the robot fulfills a leveraging role and enables the learners to succeed in a task that they might fail without the robot’s assistance.

To fulfill the teacher- as well as the learner-assisting role, the robot needs to understand the learning task at hand and its demands. It has to assess the learners’ competence, estimate their potential abilities, and monitor progress. In addition to registering overt task performance, this requires a robot to interpret multimodal cues (eye gaze, body posture, etc.) in order to infer the learner’s cognitive and emotional state and to react to situations in which the learner might struggle with a given task. To assist the learner in solving a task, the robot further needs to be able to provide appropriate cues via gestures, speech, or other means of directing attention and providing information (see also Vogt et al., 2017). Critically, to scaffold learning optimally, the robot needs to know not only *how* to help in a given context but also *when* its help is required and then balance its assistance accordingly. If the robot is too helpful this might increase short-term success but reduce the opportunity for long-term learning (for a discussion of the assistance problem, see Koedinger et al., 2008).

### 3.2 Using a social robot to offer socioemotional support to children

An emerging role for educational robots is to support children by either engaging and motivating them during a learning activity or by alleviating anxiety associated with learning a new or challenging skill. A robot can serve this role exclusively or in conjunction with other roles of being a tutor or a peer (see Section 3.1). Unlike other education robot roles, supportive robots can promote learning indirectly by optimizing the child’s socioemotional context to promote engagement in (and mitigate avoidance of) learning tasks. This could well be of significant value for children who have learning, attention, or literacy difficulties. Such supportive roles have been examined widely in healthcare settings to improve treatment adherence for chronic conditions (e.g., asthma) (Ferrante et al., 2021) and to improve mental health outcomes for isolated and infirm children (Jeong et al., 2015; Rossi et al., 2022). Crossman et al. (2018) exposed 87 children to a stressful task. Those who interacted with a social robot experienced greater reductions in stress on subjective state anxiety and salivary cortisol measures in comparison to children from two control conditions, in which either the robot was turned off or not present. However, the broader potential for social robots to simultaneously support socioemotional and education outcomes in children remains unexplored. A recent mixed-methods study by Caruana et al., (2022) explored the potential for three different robots (NAO, MiRo, and Cozmo) to support children’s engagement in reading. Whereas only one robot (NAO) had the capacity to engage in social dialogue, all robots demonstrated the potential to support children’s engagement by responding with nonspeech vocalizations, sounds, and movements (e.g., grunting, head shaking, and tail wagging). During in-depth interviews, most children reported that the robots offered a welcomed, engaging, “calming,” and nonjudgmental social context for reading. For this reason, many children expressed a preference for reading a difficult book to a robot than to either their teacher, or themselves alone. This preference was most prevalent among children who interacted with NAO. Further, children directly associated the dialogical (in)abilities of their chosen robot as a signal of its intelligence and sentience, and thus its capacity to comprehend the learning activity and assist the child if needed. As such, whilst supportive robots need not serve in dialogical capacities (e.g., Paro the nonverbal zoomorphic robot), social dialogue may help robots to assert their social presence and their capacity to fully co-experience the learning activity. Further, features of robot speech can shape a supportive learning context―particularly when robots can adapt/entrain to features of a human interlocutor’s speech (Kumar et al., 2010; Gulz et al., 2011). For example, Lubold et al., (2018) observed middle school children teaching a robot to solve ratio problems. Children experienced greater learning and social rapport when the robot engaged in social dialogue and adapted to the child’s pitch, compared to robots who engaged in social dialogue without this adaptation or no social dialogue at all. We can thus conclude that dialogical and supportive robots offer great promise for education interventions. However, fulfilling their full potential will require autonomous robots that can accurately and rapidly perceive, comprehend, and respond to child speech in the absence of adult operators. Such needs currently outstrip the capabilities of most robots and speech recognition–production systems.

The studies presented above suggest that the mere presence of “supportive” robots has the potential to improve children’s socioemotional state during difficult learning tasks (see Caruana et al., 2022, for a discussion). However, larger gains are likely to be seen if robots can actively motivate children or change the way children evaluate a learning activity and their capacity to complete it. In this respect, consider a child experiencing reading difficulty and associated anxiety. A support robot may promote active engagement simply by offering periodic prompts that encourage the child to continue reading or relay the amount of time or the number of pages already read in a session. Such capabilities would be easy to automate. It is important to highlight that for such a support, robots must be able to rapidly sense and recognize when a child is making errors and thus stops reading or becomes anxious. Then, they could offer sensitive encouragement and reinforcement *when* the child needs it, and thus encourage the child to persist. This could be accompanied by questions prompting the child to reflect on their reading success: “That was a hard one, but you read it! How do you feel?” Another future direction is to develop robots that can support reading engagement through multiple dialogical roles (e.g., a supportive co-learner) because they can also make errors themselves to elicit corrective feedback from the child (see Section 3.5, Grimminger and Rohlfing, 2017). They could also ask spontaneous comprehension questions—a successful method that is known from dialogical reading (Blewitt et al., 2009)—to promote and check the child’s attention while framing the robot as engaged, competent, but also un-intimidatingly flawed. This thus promotes engagement and mitigates apprehension. Indeed, recent work has shown that robots that can adaptively move between the roles of *tutor* or *peer/novice* can maximally support children’s learning and emotional needs during vocabulary acquisition (Chen et al., 2020). This again demonstrates that the social roles robots can adopt during learning interventions do not need to be fixed or discrete; and, indeed, dynamic, and adaptive education roles are likely to best position social robots as socioemotional supports for children engaging in learning activities. We will come to this point within the Discussion (see also Figure 1).

### 3.3 Using a social robot to nudge or prompt children’s communicative behavior

Social robots are particularly useful compared to other types of technology to prompt or nudge others’ communicative behavior. They often have a humanoid or animal-like appearance, and this increases the tendency to anthropomorphize them (Mubin et al., 2013), thus, making it more likely that people will speak to them. Lin et al. (2022) discussed oral interactions between learners and robots in their recent RALL review. One of their findings was that interactive oral tasks (such as engaging in dialogues with robots) are often used in foreign-language-learning classes—because robots provide learners with the opportunity to engage in dialogues with a “native” speaker—and these activities are aimed more at practicing communicative skills than improving grammatical accuracy. Robots are used for such communicative activities for a reason: Learners are often less anxious about engaging in dialogues with social robots than peer learners or teachers, because they feel less judged and less afraid of making mistakes (Alemi et al., 2015; see also Section 3.2). The robot may thus serve as a middle ground between the benefits of a tutor (high-quality language input and feedback) and less-anxiety-inducing environment of practicing with a peer. This does not mean, however, that robots can be used only in elaborate conversational classes with language learners who have some proficiency in the language that they speak with the robot. Robots can also be used to prompt novice learners to use expressive language. For example, children in Vogt et al. (2019) had little prior knowledge of English and were invited by the robot to repeat English target words. This study investigated a long-term effect of learning by testing children’s recall experimentally, but it did not address the question of whether children were motivated to use the learned words in their everyday context. In this sense, to prompt somebody means to provide an impulse that is taken up not only in a context that requires or elicits it—as is the case in an experimental session requiring active learning—but also in other contexts. The exploitation of the impulse in other contexts would reflect the constructive learning (see Table 2).

Facing all the tasks in which a robot prompts children in their behavior, it becomes apparent that speech recognition technology plays an important role in their design. Yet, although advancements are being made, current speech technology is still limited in recognizing speech, especially that of young children (Kennedy et al., 2017). This is probably one of the reasons for why only a few studies with novice learners invite the learner to use language expressively. Most studies include games in which learners can respond in other ways such as selecting an answer on a tablet (de Wit et al., 2018). If robots are to effectively prompt children to use language expressively, they should be able to recognize what children are saying. It would be even better if they could detect language input with such accuracy that they could correct pronunciation errors. In that case, robots could provide learners with not only a less-stressful environment in which to practice a language but also feedback on how to improve their pronunciation. High-functioning speech recognition, however, is not the full story. The ability to initiate and maintain a dialogue is still a challenge for social robots along with the ability to adjust expressions for a particular content (Conti et al., 2020).

### 3.4 Using a social robot in role plays

The term “role play” refers to a scenario in which children imagine and act out a certain role by collaboratively negotiating the plot. Scenarios can be flexibly adjusted to create reality or fiction. They provide the context for negotiating and altering meaning and thus allow for constructing common ground. Common ground refers to the shared knowledge between interlocutors that is critical in communication and that can be enriched through processing and accumulating new information in communicative interactions (Clark, 2015). Role plays have been shown to enhance metacommunicative skills and to provide adequate language learning support in the preschool years (e.g., Andresen 2005) as well as in the classroom context of foreign-language learning (Raz, 1985; Al-Arishi, 1994). It is therefore reasonable to ask whether a social robot could successfully accomplish such role plays or role-playing games. For example, the robot could take the role of a shopkeeper that interacts with a child customer, the role of a police officer identifying a shoplifter, or the role of a repair service that needs help with technical tools in order to fix a bike. The robot could serve as a model for a child who, when switching roles, could imitate behavioral and linguistic patterns (see also Carpenter et al., 2005) that are appropriate in a given scenario. The number of studies on this issue is heavily limited and covers mainly adult participants. This is true for shop scenarios in which a robot provides a service through natural interactions with adult speakers (e.g., Lee et al., 2011; Liu et al., 2018); language cafés in which learners engage in small talk with a robot who takes up different roles such as interviewer, narrator, facilitator, or interlocutor (Engwall et al., 2021); and in a role-playing scenario inspired by Game of Thrones to teach a novel language (Ali et al., 2019).

Role plays go beyond the more common conversational classes discussed in Section 2, because they are based on task-based language teaching (Ellis, 2003) and require building a social relationship between the participants in a given scenario. This relationship is achieved by producing proactive and socially appropriate behavior. Whereas the high social appropriateness of predicted and proactive behaviors (Liu et al., 2018) as well as replies to social contexts that are coordinated and timely (Belpaeme et al., 2018) are currently not implemented in robots, this could foster learning of role-related procedures. For example, the robot needs to predict what type of interaction and behavior it should evoke from a child in a given role play and to respond adequately. It should also adjust its behavior flexibly when switching roles in a given scenario. Again, the recognition of nonverbal social signals such as facial expressions, gestures, and posture is needed for the right interpretation of the situation and for appropriate replies providing hints and encouragement in a specific role.

In summary, role plays could foster vocabulary acquisition and the development of communicative competence. Following the task-based language teaching approach (Ellis, 2003), a child or a second-language learner could practice meaningful real-life verbal skills by establishing a social relationship with the robot and solving a task or pretending to do so. Finally, role plays provide an excellent opportunity for what are considered to be the best forms of teaching: activating knowledge, boosting meaning negotiations, and the co-construction of knowledge (Chi, 2009). Such activities could be particularly relevant for learners from diverse cultural backgrounds, who could practice conversations in the new language before having them with actual speakers of that language. Future studies could examine the effectiveness of a robot in meaningful role-play interactions and learning gains as a function of the design, functionalities of the robot, and most importantly the individual characteristics of the child interacting with the robot.

### 3.5 Using an incorrect social robot to promote reflection and error correction

Since the late 1960s, when researchers made the surprising discovery that, in a peer tutoring setting, peer tutors progressed more than their own tutees (Cloward, 1967), literature has extensively investigated the benefits of “learning-by-teaching” interactions (Duran, 2017). Learning with the expectation to teach was found to promote the identification of key content elements and their organization in a meaningful representation (Benware and Deci, 1984), whereas “learning and explaining” was found to allow for more persistent learning gains than learning with the expectation to teach (Fiorella and Mayer, 2013). Explaining to others provides the tutor with more and better opportunities to recognize own areas for improvement, reorganize their own knowledge, and repair their own errors by exercising their metacognitive skills (Duran, 2017).

In an effort to provide learners with more opportunities for engaging in “learning-by-teaching” interactions, teachable virtual agents (Biswas et al., 2005) and teachable robots (Tanaka and Matsuzoe, 2012; Hood et al., 2015; Walker et al., 2016) have been developed. Within the latter category, an intriguing research avenue is to explore the effect that purposefully designed incorrect behaviors of a robot have on the engagement and learning of its human tutor. With numerous studies in the field of human–robot interaction supporting the fact that faulty robots are consistently perceived as more likeable than their infallible alternatives (Ragni et al., 2016; Mirnig et al., 2017), it seems plausible to argue that incorrect^2^ teachable robots further reduce the learners’ anxiety, making them feel even less judged and afraid of making mistakes (Alemi et al., 2015). Children’s promptness at adopting a care-taking attitude toward social robots showing weaknesses (Tanaka and Matsuzoe, 2012) seems particularly apt; and, in addition, children’s motivation and engagement as tutors speaks to the “protégé effect,” which seems to especially benefit lower-achieving students (Chase et al., 2009). Finally, incorrect behaviors displayed by the robot can 1) be the trigger for a correction spontaneously provided by the child (thus providing a natural framing for a “learning-by-teaching” interaction), and 2) via a careful design of the robot’s behavior, highlight specific errors and thus nudge the child towards specific corrections. These last two benefits, uniquely brought by incorrect teachable robots, have been investigated by two studies.

In a scenario aiming to teach English verbs to 3- to 6-year-old Japanese children, a NAO robot was used in a “learning-by-teaching” interaction that envisioned a first phase in which an adult teacher taught both the child and the robot, followed by a phase in which the child could revise the robot’s initially wrong understanding and correct it (Tanaka and Matsuzoe, 2012). Results indicated a higher learning gain compared to a control group of children not interacting with the robot in both an immediate and a delayed post-test administered 3–5 weeks after the experiment. Interestingly, parents reported that their children liked the experience of teaching the robot so much that they continued to play it at home—even days and weeks after the experiment—thus suggesting that the interaction with the robot could have promoted their spontaneous learning. In this study, the robot’s initially incorrect behavior was used as a trigger and motivator for the “learning-by-teaching” interaction. It would be interesting to compare such a robot with a “traditional” robot tutee to verify the effects of the robot’s mistakes on the children’s engagement in the interaction and learning.

In another study known as CoWriter (Hood et al., 2015; Chandra et al., 2017), a NAO robot with poor handwriting skills was used in a “learning-by-teaching” interaction with children to stimulate their metacognition, empathy, and self-esteem in addition to their handwriting skills. The interaction consisted of the following sequence: The child first selects the letter to help NAO practice on. The robot then “writes” it on a digital tablet (concretely, moving its finger in front of the tablet to follow the letter’s trajectory). Finally, it asks for feedback as well as an example from the child and incorporates this in its next attempt to write the letter. It continues its attempts until the child deems the result to be satisfactory. Beside incorporating the child’s feedback, at each iteration the robot’s letter includes a deformation in proportion, breaks and/or alignment that Chandra and colleagues (2017) identified as categories of mistakes commonly performed by children. Helping the robot improve its handwriting thus induced the children to reflect on common handwriting mistakes and how to correct them. In 2018–2019, the CoWriter setup was integrated into the weekly occupational therapy sessions for a 10-years old boy with a complex neurodevelopmental disorder including severe dysgraphia, for whom previous therapies had not led to noticeable improvements (Gargot et al., 2021). After 20 sessions, the boy’s handwriting skills had improved significantly, and a decrease in avoidance behaviors as well as better commitment to handwriting practice could be observed. As a consequence, he could go back to a regular school where he received special education. Interestingly, although the boy reflected on the intentions behind the robot’s behavior (“It is not the robot who learns, it is me” Gargot et al., 2021, *p*. 6) relatively early in the intervention, he kept tutoring the robot and engaging with it throughout the sessions.

Unfortunately, the tremendous potential of incorrect teachable robots, which combines the advantages brought by “learning-by-teaching” interaction with the unique engagement and metacognition boost provided by the protégé effect inspired by the robot’s shortcomings, comes at a high technical cost. Quite ironically, a good “bad-performing” robot is possibly even more difficult to design than a good “well-performing” robot. What seems particularly difficult is to concurrently and consistently ensure that 1) the robot’s incorrect behavior triggers the desired effects in terms of engagement and metacognition without generating frustration in the human tutor (Biswas et al., 2005), 2) the robot improves over time, thus incorporating the tutor’s scaffolding behavior and making it interactive (Chi, 2009) while 3) never surpassing the tutor’s own competence, which would negatively impact the tutor’s self-esteem. Tanaka and Matsuzoe (2012) circumvented this problem by remotely tele-operating the robot with a Wizard-of-Oz approach, whereas Hood et al. (2015) and Chandra et al. (2017) relied on a dataset of adult handwriting samples to define shape deformations to apply to the letters’ models, thereby ensuring the robot’s “bad handwriting.” Upon merging the robot’s own poor letter with the example provided by the child, errors are either mitigated (if they do not appear in the example) or reinforced: This enables the child to see their own mistakes in the robot’s handwriting and reflect on them. Exporting such a sophisticated interaction to more complex contexts, possibly involving social and verbal interaction, is an open research challenge.

### 3.6 Using a social robot to encourage metatalk in children

A dialogical role that has received little attention in current implementations of social robots is that of a robot encouraging metatalk. This term encompasses both the ability to talk about communication in general (metacommunication) or language in particular (metalinguistics). In the case of communication, there can be talk about organizing the ongoing interaction or about hypothetical conversations; in the case of language, the talk can be about its structure (Aukrust, 2004). In both cases, communication becomes the focus of the communicative activity, is manipulated, or is reflected upon. To date, there has been hardly any focus on this ability in child–robot interaction studies on language learning, or it is usually addressed only implicitly as in studies on improving children’s narrative abilities (e.g., Kory Westlund and Breazeal, 2019). To reflect on communication is an ability relying on metacognition. Metacognition, in turn, can be defined as “awareness and management of one’s own thought” (Kuhn and Dean, 2004, *p*. 270) and is driven by executive control.

Considering metalinguistic abilities as foundational for a child’s communicative competence and literacy outcomes (Heller, 2014; Stude, 2014), Hsiao et al., (2015) carried out a study in which a robot interacted with a pair of two kindergarten children in a book-reading situation in which the children were requested to reflect on sentence patterns and compare words. The robot was equipped with the ability to produce speech and sounds as well as to automatically provide emotional responses to the children’s turns via its integrated touchscreen. Interestingly, in addition to improving children’s overall reading skills, the authors reported that opportunities arose for children to share reflections about the meaning of the text, such as one child explaining the meaning of a word to the other or both discussing parts over which one of them disagreed (Hsiao et al., 2015, *p*. 287). Furthermore, and with regard to the reading abilities necessary to solve math problems, Ramachandran et al. (2018) applied a robot that encouraged 11-year-old children to think aloud—a metacognitive strategy. Their results indicated positive effects of a social robot on the students’ engagement and compliance with the proposed thinking-aloud strategy. The authors concluded that a robot can support the use of a metacognitive strategy to enhance problem solving in children.

In addition, recently, Spaulding and colleagues (2021) used a robot as a learning companion to play language games with children. It engaged them in activities of spelling and rhyming words in order to promote their phonological awareness, i.e., knowledge about sounds in their spoken language (Spaulding et al., 2021). The contextual environment was displayed on a tablet, and by taking turns with the child, the robot performed game tasks and responded to the child’s input on the tablet with socioemotional behaviors. In addition, the robot modeled the learner’s behavior and “demonstrated” exemplary tasks to the child based on the child’s play actions and state of knowledge (Spaulding et al., 2021, *p*. 5).

Taken together, these ideas highlight a clear potential for the use of robots in the dialogical role of initiating metatalk in children—albeit the aforementioned studies focus mainly on talk about language in particular rather than metaknowledge about dialogue. Additionally, in terms of Chi’s (2009) proposed categories of engagement, further interactive capabilities are needed. Whereas the approaches presented here can be considered primarily as actively and constructively eliciting children’s elaborations and reflections that go beyond the immediate learning content presented, future empirical studies should move toward an interactive learning. Specifically, interaction scenarios aiming to enable children to talk about dialogical features such as peculiarities experienced within the turn-taking with a robot that takes longer to react (Tolksdorf et al., 2021), could elicit reflections on dialogical processes in general, and turn-taking in particular: Children could reflect on how long a pause between a question and its answer could be and what reactions a too long pause elicits. These reflections could help children to develop coping strategies for peculiarities or idiosyncrasies within a dialogue without referring to persons (Horwath et al., 2018). Overall, there seems to be an inexhaustible potential for using social robots to support metatalk in children.

## 4 Discussion

In this review, we have proposed innovative roles that a social robot can fulfill in an interaction with a child. This extends established roles that enable a social robot to act as a tutor, peer/companion, or novice/learner (Mubin et al., 2013; Ahmad et al., 2021; Lin et al., 2022) through being less rigid and more fluid in relation to the established role categories (see Figure 1). At the same time, these roles offer innovative forms of education for learning language and literacy that further differentiate the existing categories with respect to domain knowledge and social competence (see Figure 1). In addition to our review, we have contributed an analytical framework that can be used to specify the differences in human–robot social relationships when children learn language through interacting with a social robot. Based on the theoretical account proposed by Mead (1946) suggesting that social roles shape interactive acts, we identified the overt acts that we then subjected to a critical evaluation reflecting the different human–robot social relationships that arise when applying a robot in a particular social role.

Identifying the skills that enable a robot to perform overt acts is a crucial step in our analysis of innovative roles. In Table 1, we differentiated between perceptual, cognitive, and dialogical skills that are required from a robot to fulfill a role. This differentiation can be seen as problematic, because cognitive and dialogical skills are intertwined. However, whereas cognitive skills are covert, dialogical behaviors are overt and easy to assess. In addition, overt behaviors are based more on pragmatic skills, and they result in decisions on choosing, for example, what formulation is appropriate for a situation. While analyzing the established roles for the skills they require, we realized that they are too coarse-grained to clearly identify the skills. Summarizing the innovative roles in Table 1, we have to highlight the fact that the majority of them requires skills that have still hardly ever been implemented in robots. Instead, both cognitive and dialogical skills are pre-programmed, and in most studies, semi-implementation of such skills is realized by applying the Wizard-of-Oz-method.

After having gained a clearer picture on the robot’s skills as prerequisites to a social role, we drew on the abilities specified in Table 1 to analyze different types of overt activities that a robot could initiate and thus use to engage with a learner (Table 2). Our analysis is guided by a taxonomy suggested by Chi (2009) who differentiated between active, constructive, and interactive activities. Providing empirical support for deeper learning being scaffolded more by interactive rather than constructive or active activities, we specified in Table 2 whether and with what behaviors the three types of learning activities can be realized when the robot is fulfilling a specific role. It becomes clear that “interactive learning” requires more responsive behavior in a robot than “active learning” does. It is important to note that this responsivity goes beyond simple contingency (Cangelosi et al., 2010) and requires more semantics. In this respect, McGillion et al. (2013) suggest the term “semantic contingency” to highlight the meaningful action that can follow on from a behavior. Because, given the current state of the art, robots are not able to understand the actions of their partners, they cannot select behavior that is both temporally and semantically contingent (such as providing not only feedback but appropriate feedback)—behavior that would better relate to the action that the partner has just performed. Consequently, a form of memory for the interaction would be necessary to enable a robot to perform more activities linked to interactive learning. With such a memory for the “history of interaction” (Rohlfing et al., 2016, *p*. 4), a robot could monitor the child’s engagement in order to note deviations, and then provide hints, encouragement, or reassurance.

When reflecting on the educational potential of the innovative social roles, we noted that most of them (see Sections 3.1–3.5) require a robot to react to children’s multimodal communicative behavior. Interestingly, despite plenty of evidence suggesting that a robot needs to react contingently and multimodally when interacting with children (Belpaeme et al., 2018), the dialogue situation is often mediated via the use of a tablet, and this restricts the child’s behavior to some choices on a screen (Vogt et al., 2017). From a technical point of view, this compensates for the robot’s inability to react to the variability in children’s communicative behavior. Yet, considering the design of interaction, this compensation clearly limits the richness of information. For example, Tolksdorf and Mertens (2020) found that children take much longer pauses when answering to a robot in which they make use of gestures and gaze rather than verbal behavior. Current robots cannot yet cope with such reactions, often resulting in interaction breaking down (Rohlfing et al., 2021). What robots thus currently lack is a system that takes full advantage of the robot’s body behaving contingently and adapting to both individual behavioral patterns and children’s speech (Kennedy et al., 2017). As long as such a dialogue system is not implemented, robotic technology will lack the interaction capabilities crucial for communication with children.

Finally, it is important to emphasize that not only can a robot appear in one role or the other, but that it can also change roles just like children do in their (learning) interactions. A change in dialogical roles—for example from being tutee to a tutor—is likely to boost learning in children by providing them with both perspectives. As discussed in Section 3.5, tutoring can be a more valuable learning experience than being tutored; the question of whether fulfilling one role and then changing to the other could advance learning even more has barely been addressed in research so far. This pertains to both studies on child–robot interaction as well as studies on child development. Among the former, Chen et al. (2020) recently proposed a robot capable of a role adaptation. Furthermore, these authors demonstrated that an adaptive role in which a robot shifts between tutor and peer/novice can lead to both learning and emotional support (see also Lee et al., 2019). Hence, role adaptation clearly brings about new possibilities to enhance learning processes. Whereas we are not aware of further studies systematically investigating the advantage of a role change for language and literacy learning, there is some discussion on the phenomenon of role reversal. More specifically, in studies with a gray parrot acquiring labels, Pepperberg (2002) argued that for the animal to learn, it was important to observe the roles of a tutor and tutee being changed and thus modeled independently from the persons. In developmental studies, Carpenter and colleagues (2005) have argued that role reversal is important for children to recognize the reciprocity of linguistic symbols. A role reversal is defined by the child performing “an action toward an adult in the same way that the adult performed it toward him or her” (Carpenter et al., 2005, *p*. 254). This enables the child to learn that there is a “reciprocal substitution between demonstrator and learner” (*p*. 255). In their study, Carpenter et al. (2005) found positive correlations between the amount of role reversal imitation and the children’s comprehension and production of pronouns. These studies make it plausible to argue that children’s learning can be advanced by a role reversal. For the design of a robot, however, it is a challenge to provide the capabilities required for a particular role, in addition to being able to reverse the interaction protocol. Chen et al. (2020) solved this by reinforcement learning and concluded that “an adaptive, reciprocal peer is more engaging, interesting, and fun for children.” However, a fine-grained analysis of the learning activities would be necessary to further evaluate the exchange, to determine whether the obtained effect is driven by more interactions (or more variability in them) being possible and investigate how long-lasting the positive effect is.

In a similar vein regarding role adaptation, we need to highlight that our focus was on the identification of the (innovative) roles for the purpose of analyzing them further. This approach sheds a rather distinguished light on the roles. However, because social interaction is dynamic in time, the roles can be fluid or change. For example, the robot in second-language studies by Vogt et al. (2017), Vogt et al. (2019) was applied in several learning sessions. Initially, children had little prior knowledge of English vocabulary. Thus, in the first session, the robot can be clearly considered to be a tutor. However, in further sessions, children become more knowledgeable advancing the robot to be a peer rather than a tutor. Whereas this example refers to a change in robot’s role from one learning session to another, a change can also take place during one session, when interaction partners become more familiar with each other. This familiarization process, in turn, is influenced by individual differences in children (Tolksdorf et al., 2021b; Konijn et al., 2022). These plausible dynamics are barely in focus in current research, nor in our review.

The framework we have presented here for a systematic analysis of the dialogical roles along with the innovative roles is only a first step toward exemplifying the potentials of dialogical roles that we consider to be crucial for learning language and literacy. We argue that these consist of perceptual, cognitive, and dialogical capabilities that should be designed depending on what kind of interaction (active, constructive, or interactive) is needed to achieve a specific educational purpose. Because many other educational domains are based on language, we are optimistic that our framework would generalize beyond language learning and literacy. Our proposed framework opens up not only many possibilities to apply dialogical roles in learning settings but also to decide on which specific role fit the purpose in order to advance language and literacy via child–robot interactions.

## Footnotes

1 We thank a reviewer for this idea.

2 We propose the terms “incorrect behavior” and “incorrect teachable robots” to denote, respectively, a robot behavior purposefully designed to include mistakes and the social robot exhibiting such behavior in the context of a “learning-by-teaching” interaction. We use this term to distinguish from “faulty robots,” whose mistakes are not necessarily planned for, while still emphasizing the nonoptimality of their actions.

## References

[B1] Admoni H. Scassellati B. (2014). “Roles of robots in socially assistive applications,” in Proceedings of the IROS workshop on rehabilitation and assistive robotics.

[B2] Ahmad M. I. Adeola E. S. Mubin O. (2021). “Towards the applicability of the social robot in the role of an invigilator,” in Proceedings of the 9th international conference on human-agent interaction, 294–299. 10.1145/3472307.3484665

[B3] Al-Arishi A. Y. (1994). Role-play, real-play, and surreal-play in the ESOL classroom. ELT J. 48 (4), 337–346. 10.1093/elt/48.4.337

[B4] Alemi M. Meghdari A. Basiri N. Taheri A. (2015). The effect of applying humanoid robots as teacher assistants to help Iranian autistic pupils learn English as a foreign language. Proc. Int. Conf. Soc. Robotics, 1–10. 10.1007/978-3-319-25554-5_1

[B5] Ali H. Bhansali S. KöksalMöller I. M. Möller M. Pekarek-Rosin T. Sharma S. (2019). “Virtual or physical? Social robots teaching a fictional language through a role-playing game inspired by game of Thrones,” in *Social robotics*. (=Lecture notes in artificial intelligence 11876). Editor Salichs M. A. (Cham: Springer), 358–367. Game of Thrones. 10.1007/978-3-030-35888-4_33

[B6] Andresen H. (2005). Role play and language development in the preschool years. Cult. Psychol. 11 (4), 387–414. 10.1177/1354067X05058577

[B7] Aukrust V. G. (2004). Talk about talk with young children: Pragmatic socialization in two communities in Norway and the US. J. Child. Lang. 31 (1), 177–201. 10.1017/S0305000903005907 15053089

[B8] Baxter P. Ashurst E. Read R. Kennedy J. Belpaeme T. (2017). Robot education peers in a situated primary school study: Personalisation promotes child learning. PloS one 12 (5), e0178126. 10.1371/journal.pone.0178126 28542648 PMC5441605

[B9] Belpaeme T. Kennedy J. Ramachandran A. Scassellati B. Tanaka F. (2018). Social robots for education: A review. Sci. Robot. 3, eaat5954. 10.1126/scirobotics.aat5954 33141719

[B10] Benware C. A. Deci E. L. (1984). Quality of learning with an active versus passive motivational set. Am. Educ. Res. J. 21 (4), 755–765. 10.3102/00028312021004755

[B11] Biswas G. Leelawong K. Schwartz D. Vye N. (2005). amp; The Teachable Agents Group at VanderbiltLearning by teaching: A new agent paradigm for educational software. Appl. Artif. Intell. 19 (3–4), 363–392. 10.1080/08839510590910200

[B12] Blewitt P. Rump K. M. Shealy S. E. Cook S. A. (2009). Shared book reading: When and how questions affect young children's word learning. J. Educ. Psychol. 101 (2), 294–304. 10.1037/a0013844

[B13] Breazeal C. Harris P. L. DeSteno D. Kory Westlund J. M. Dickens L. Jeong S. (2016). Young children treat robots as informants. Top. Cogn. Sci. 8 (2), 481–491. 10.1111/tops.12192 26945492

[B14] Broekens J. Heerink M. Rosendal H. (2009). Assistive social robots in elderly care: A review. Gerontechnology 8 (2), 94–103. 10.4017/gt.2009.08.02.002.00

[B15] Cangelosi A. Metta G. Sagerer G. Nolfi S. Nehaniv C. FischerTani K. J. (2010). Integration of action and language knowledge: A roadmap for developmental robotics. IEEE Trans. Auton. Ment. Dev. 2, 167–195. 10.1109/tamd.2010.2053034

[B16] Carpenter M. Tomasello M. Striano T. (2005). Role reversal imitation and language in typically developing infants and children with autism. Infancy 8, 253–278. 10.1207/s15327078in0803_4

[B17] Caruana N. Moffat R. Blanco A. M. Cross E. S. (2022). Perceptions of intelligence &amp; sentience shape children's interactions with robot reading companions: A mixed methods study. PsyArXiv. 10.31234/osf.io/7t2w9 PMC10162967 37147422

[B18] Chandra S. Dillenbourg P. Paiva A. (2017). “Classification of children's handwriting errors for the design of an educational co-writer robotic peer,” in Proceedings of the conference on interaction design and children, 215–225. 10.1145/3078072.3079750

[B19] Chase C. C. Chin D. B. Oppezzo M. A. Schwartz D. L. (2009). Teachable agents and the protégé effect: Increasing the effort towards learning. J. Sci. Educ. Technol. 18 (4), 334–352. 10.1007/s10956-009-9180-4

[B20] Chen H. Park H. W. Breazeal C. (2020). Teaching and learning with children: Impact of reciprocal peer learning with a social robot on children's learning and emotive engagement. Comput. Educ. 150, 103836. 10.1016/j.compedu.2020.103836

[B21] Chi M. T. (2009). Active-constructive-interactive: A conceptual framework for differentiating learning activities. Top. Cognitive Sci. 1 (1), 73–105. 10.1111/j.1756-8765.2008.01005.x 25164801

[B22] Clark E. (2015). “Common ground,” in The handbook of language emergence. Editors MacWhinney B. O’Grady W. (London: Wiley-Blackwell), 328–353. 10.1002/9781118346136.ch15

[B23] Cloward R. D. (1967). Studies in tutoring. J. Exp. Educ. 36 (1), 14–25. 10.1080/00220973.1967.11011022

[B24] Conti D. Cirasa C. Di Nuovo S. Di Nuovo A. (2020). "Robot, tell me a tale!". Is 21 (2), 220–242. 10.1075/is.18024.con

[B25] Crossman M. K. Kazdin A. E. Kitt E. R. (2018). The influence of a socially assistive robot on mood, anxiety, and arousal in children. Prof. Psychol. Res. Pract. 49 (1), 48–56. 10.1037/pro0000177

[B26] Cumbal R. (2022). “Adaptive robot discourse for language acquisition in adulthood,” in Proceedings of the ACM/IEEE international conference on human-robot interaction (IEEE), 1158–1160.

[B27] Čaić M. Mahr D. Oderkerken-Schröder G. (2019). Value of social robots in services: Social cognition perspective. J. Serv. Mark. 33 (4), 463–478.

[B28] de Graaf M. (2016). An ethical evaluation of human-robot relationships. Int J Soc Robotics 8 (4), 589–598. 10.1007/s12369-016-0368-5

[B29] de Wit J. Schodde T. Willemsen B. Bergmann K. de Haas M. Kopp S. (2018). “The effect of a robot’s gestures and adaptive tutoring on children’s acquisition of second language vocabularies,” in Proceedings of the ACM/IEEE international conference on human-robot interaction (IEEE), 50–58.

[B30] Deublein A. Pfeifer A. Merbach K. Bruckner K. Mengelkamp C. Lugrin B. (2018). Scaffolding of motivation in learning using a social robot. Comput. Educ. 125, 182–190. 10.1016/j.compedu.2018.06.015

[B31] Duran D. (2017). Learning-by-teaching. Evidence and implications as a pedagogical mechanism. Innovations Educ. Teach. Int. 54 (5), 476–484. 10.1080/14703297.2016.1156011

[B32] Ellis R. (2003). Task-based language learning and teaching. Oxford: Oxford University Press.

[B33] Engwall O. Lopes J. Åhlund A. (2021). Robot interaction styles for conversation practice in second language learning. Int. J. Soc. Robotics 13, 251–276. 10.1007/s12369-020-00635-y

[B34] Engwall O. Lopes J. (2022). Interaction and collaboration in robot-assisted language learning for adults. Computer Assisted Language Learning 35 (5–6), 1273–1309.

[B35] Ferrante G. Vitale G. Licari A. Montalbano L. Pilato G. Infantino I. (2021). Social robots and therapeutic adherence: A new challenge in pediatric asthma? Paediatr. Respir. Rev. 40, 46–51. 10.1016/j.prrv.2020.11.001 33386280

[B36] Fiorella L. Mayer R. E. (2013). The relative benefits of learning by teaching and teaching expectancy. Contemp. Educ. Psychol. 38 (4), 281–288. 10.1016/j.cedpsych.2013.06.001

[B37] Gargot T. Asselborn T. Zammouri I. Brunelle J. Johal W. Dillenbourg P. (2021). "It is not the robot who learns, it is me." treating severe dysgraphia using child-robot interaction. Front. Psychiatry 12, 596055. 10.3389/fpsyt.2021.596055 33716812 PMC7950539

[B38] Gordon G. Breazeal C. (2015). “Bayesian active learning-based robot tutor for children’s word-reading skills,” in Proceedings of the twenty-ninth AAAIConference on artificial intelligence, 1343–1349.

[B39] Grimminger A. Rohlfing K. J. (2017). “6th workshop on child computer interaction (WOCCI 2017),” in Proceedings of the 6th international workshop on child computer interaction (WOCCI 2017), 28–33. 10.21437/WOCCI.2017

[B40] Gulz A. Haake M. Silvervarg A. (2011). “Extending a teachable agent with a social conversation module - effects on student experiences and learning,” in Artificial intelligence in education. Editors Biswas G. Bull S. Kay J. Mitrovic A. (Springer Berlin Heidelberg), 6738, 106–114. 10.1007/978-3-642-21869-9_16

[B41] Han J. Kang B. Park S. Hong S. (2012). “How to sustain long-term interaction between children and ROBOSEM in English class,” in Proceedings of the seventh annual ACM/IEEE international conference on human-robot interaction, 421–422. 10.1145/2157689.2157828

[B42] Heller V. (2014). Discursive practices in family dinner talk and classroom discourse: A contextual comparison. Learn. Cult. Soc. Interact. 3 (2), 134–145. 10.1016/j.lcsi.2014.02.001

[B43] Hemminghaus J. Koop S. (2017). “Towards adaptive social behavior generation for assistive robots using reinforcement learning,” in Proceedings for the 2017 ACM/IEEE international conference on human-robot interaction, 332–340. 10.1145/2909824.3020217

[B44] Hong Z.-W. Huang Y.-M. Hsu M. Shen W.-W. (2016). Authoring robot-assisted instructional materials for improving learning performance and motivation in EFL classrooms. J. Educ. Techno. Soc. 19 (1), 337–349.

[B45] Hood D. Lemaignan S. Dillenbourg P. (2015). “When children teach a robot to write: An autonomous teachable humanoid which uses simulated handwriting,” in Proceedings of the tenth annual ACM/IEEE international conference on human-robot interaction, 83–90.

[B46] Horwath I. Kolossa D. Rohlfing K. J. Wrede B. Zorn I. (2018). “CRICKET: Critical technological thinking in early Education,” in Presented at the Auswahlkolloquium der VolkwagenStiftung im Rahmen der Förderinitative “Künstliche Intelligenz—ihre Auswirkungen auf die Gesellschaft von morgen (Hannover, 15. Oktober 2018.

[B47] Hsiao H.-S. Chang C.-S. Lin C.-Y. Hsu H.-L. (2015). "iRobiQ": The influence of bidirectional interaction on kindergarteners' reading motivation, literacy, and behavior. Interact. Learn. Environ. 23 (3), 269–292. 10.1080/10494820.2012.745435

[B48] Jeong S. Logan D. E. Goodwin M. S. Graca S. O'Connell B. Goodenough H. (2015). A social robot to mitigate stress, anxiety, and pain in hospital pediatric care. Proc. Tenth Annu. ACM/IEEE Int. Conf. Human-Robot Interact. Ext. Abstr., 103–104. 10.1145/2701973.2702028

[B49] Kanda T. Hirano T. Eaton D. Ishiguro H. (2004). Interactive robots as social partners and peer tutors for children: A field trial. Human-Comp. Interact. 19, 61–84. 10.1207/s15327051hci1901&2_4

[B50] Kanero J. Geçkin V. Oranç C. Mamus E. Küntay A. C. Göksun T. (2018). Social robots for early language learning: Current evidence and future directions. Child. Dev. Perspect. 12 (3), 146–151. 10.1111/cdep.12277

[B51] Kennedy J. Lemaignan S. Montassier C. Lavalade P. Irfan B. Papadopoulos F. (2017). “Child speech recognition in human-robot interaction: Evaluations and recommendations,” in Proceedings of the ACM/IEEE international conference on human-robot interaction (IEEE), 82–90.

[B52] Koedinger K. R. Pavlik P. McLaren B. M. Aleven V. (2008). “Is it better to give than to receive? The assistance dilemma as a fundamental unsolved problem in the cognitive science of learning and instruction,” in Proceedings of the 30th meeting of the cognitive science society, 1–6.

[B53] Konijn E. A. Jansen B. Mondaca Bustos V. Hobbelink V. L. N. F. Preciado Vanegas D. (2022). Social robots for (second) language learning in (migrant) primary school children. Int J Soc Robotics 14, 827–843. 10.1007/s12369-021-00824-3

[B54] Kory Westlund J. M. K. Martinez M. Achie M. Das M. Breazeal C. (2016). “Effects of framing a robot as a social agent or as a machine on children’s social behavior,” in 25^th^ IEEE international symposium on robot and human interactive communication (RO-MAN), 688–693.

[B55] Kory-Westlund J. M. Breazeal C. (2019). A long-term study of young children's rapport, social emulation, and language learning with a peer-like robot playmate in preschool. Front. Robot. AI 6, 81. 10.3389/frobt.2019.00081 33501096 PMC7806079

[B56] Kuhn D. Dean, Jr. D. Jr (2004). Metacognition: A bridge between cognitive psychology and educational practice. Theory Into Pract. 43 (4), 268–273. 10.1207/s15430421tip4304_4

[B57] Kumar R. Ai H. Beuth J. L. Rosé C. P. (2010). “Socially capable conversational tutors can Be effective in collaborative learning situations,” in Intelligent tutoring systems. Editors Aleven V. Kay J. Mostow J. (Springer Berlin Heidelberg), 6094, 156–164. 10.1007/978-3-642-13388-6_20

[B58] Lee H. Lee J. H. (2022). The effects of robot-assisted language learning: A meta-analysis. Educ. Res. Rev. 35, 100425. 10.1016/j.edurev.2021.100425

[B59] Lee M. Ackermans S. Van As N. Chang H. Lucas E. IJsselsteijn W. (2019). “Caring for vincent: A chatbot for self-compassion,” in Proceedings of the CHI conference on human factors in computing systems. 1–13.

[B60] Lee S. Noh H. Lee J. Lee K. Lee G. Sagong S. (2011). On the effectiveness of robot-assisted language learning. ReCALL 23 (1), 25–58. 10.1017/10.1017/s0958344010000273

[B61] Lin V. Yeh H. C. Chen N. S. (2022). A systematic review on oral interactions in robot-assisted language learning. Electronics 11 (2), 290. 10.3390/electronics11020290

[B62] Liu P. Glas D. F. Kanda T. Ishiguro H. (2018). Learning proactive behavior for interactive social robots. Auton. Robot. 42, 1067–1085. 10.1007/s10514-017-9671-8

[B63] Lubold N. Walker E. Pon-Barry H. Ogan A. (2018). “Automated pitch convergence improves learning in a social, teachable robot for middle school mathematics,” in Proceedings of the international conference on artificial intelligence in education (Cham: Springer), 282–296. 10.1007/978-3-319-93843-1_21

[B64] McGillion M. L. Herbert J. S. Pine J. M. Keren-Portnoy T. Vihman M. M. Matthews D. E. (2013). Supporting early vocabulary development: What sort of responsiveness matters? IEEE Trans. Auton. Ment. Dev. 5 (3), 240–248. 10.1109/tamd.2013.2275949

[B65] Mead G. H. (1946). From the standpoint of a social behaviorist. Chicago, IL: The University of Chicago Press. Mind, self & society

[B66] Mirnig N. Stollnberger G. Miksch M. Stadler S. Giuliani M. Tscheligi M. (2017). To err is robot: How humans assess and act toward an erroneous social robot. Front. Robot. AI 4, 21. 10.3389/frobt.2017.00021

[B67] Movellan J. R. Eckhardt M. Virnes M. Rodriguez A. (2009). “Sociable robot improves toddler vocabulary skills,” in Proceedings of the 4th ACM/IEEE international conference on human-robot interaction, 307–308. 10.1145/1514095.1514189

[B68] Mubin O. Stevens C. J. Shahid S. Mahmud A. A. Dong J.-J. (2013). A review of the applicability of robots in education. Techology Educ. Learn. 1, 1–7. 10.2316/journal.209.2013.1.209-0015

[B69] Neumann M. M. (2020). Social robots and young children's early language and literacy learning. Early Child. Educ. J. 48, 157–170. 10.1007/s10643-019-00997-7

[B70] Newhart V. A. Warschauer M. Sender L. S. (2016). Virtual inclusion via telepresence robots in the classroom: An exploratory case study. Int. J. Technol. Learn. 23 (4), 9–25. 10.18848/2327-0144/cgp/v23i04/9-25

[B71] Norman U. Bruno B. Dillenbourg P. (2021). “Mutual modelling ability for a humanoid robot: How can it improve my learning as we solve a problem together?,” in Robots for learning workshop in 16th annual IEEE/ACM conference on human-robot interaction (HRI 2021), 1–6.

[B72] Oranç C. Küntay A. C. (2020). Children's perception of social robots as a source of information across different domains of knowledge. Cogn. Dev. 54, 100875. 10.1016/j.cogdev.2020.100875

[B73] Pepperberg I. M. (2002). The alex studies: cognitive and communicative abilities of grey parrots. Cambridge, MA: Harvard University Press.

[B74] Ragni M. Rudenko A. Kuhnert B. Arras K. O. (2016). “Errare humanum est: Erroneous robots in human-robot interaction,” in 25th IEEE international symposium on robot and human interactive communication (RO-MAN) (IEEE), 501–506. 10.1109/roman.2016.7745164

[B75] Ramachandran A. Huang C. M. Gartland E. Scassellati B. (2018). “Thinking aloud with a tutoring robot to enhance learning,” in Proceedings of the ACM/IEEE international conference on human-robot interaction (HRI) (IEEE), 59–568. 10.1145/3171221.3171250

[B76] Raz H. (1985). Role-play in foreign language learning. System 13 (3), 225–229. 10.1016/0346-251X(85)90037-5

[B77] Rohlfing K. J. Grimminger A. Wrede B. (2021). “The caregiver’s role in keeping a child–robot interaction going,” in International perspectives on digital media and early literacy: The impact of digital devices on learning, language acquisition, and social interaction. Editors Rohlfing K. J. Müller-Brauers C. (London and New York: Routledge), 73–89.

[B78] Rohlfing K. J. Wrede B. Vollmer A. L. Oudeyer P. Y. (2016). An alternative to mapping a word onto a concept in language acquisition: Pragmatic frames. Front. Psychol. 7, 470. 10.3389/fpsyg.2016.00470 27148105 PMC4835869

[B79] Rossi S. Santini S. J. Di Genova D. Maggi G. Verrotti A. Farello G. (2022). Using the social robot NAO for emotional support to children at a pediatric emergency department: Randomized clinical trial. J. Med. Internet Res. 24 (1), e29656. 10.2196/29656S0958344010000273 34854814 PMC8796042

[B80] Schodde T. Hoffmann L. Stange S. Kopp S. (2019). Adapt, explain, engage—a study on how social robots can scaffold second-language learning of children. ACM Trans. Human-Robot Interact. (THRI) 9 (1), 1–27.

[B81] Sharkey A. J. (2016). Should we welcome robot teachers? Ethics Inf. Technol. 18 (4), 283–297. 10.1007/s10676-016-9387-z

[B82] Shin N. Kim S. (2007). “Learning about, from, and with robots: Student’ perspectives,” in Proceedings of the RO-MAN 2007-the 16th IEEE international symposium on robot and human interactive communication (IEEE), 1040–1045.

[B83] Spaulding S. Chen H. Ali S. Kulinski M. Breazeal C. (2018). “A social robot system for modeling children’s word pronunciation,” in Proceedings of the seventeenth international conference on autonomous agents and multiagent systems, 1658–1666.

[B84] Spaulding S. Shen J. Park H. W. Breazeal C. (2021). Lifelong personalization via Gaussian process modeling for long-term HRI. Front. Robot. AI 8, 683066. 10.3389/frobt.2021.683066 34164437 PMC8215502

[B85] Stude J. (2014). The acquisition of discourse competence: Evidence from preschoolers' peer talk. Learn. Cult. Soc. Interact. 3 (2), 111–120. 10.1016/j.lcsi.2014.02.006

[B86] Tanaka F. Matsuzoe S. (2012). Children teach a care-receiving robot to promote their learning: Field experiments in a classroom for vocabulary learning. Jhri 1 (1), 78–95. 10.5898/jhri.1.1.tanaka

[B87] Tolksdorf N. F. Crawshaw C. E. Rohlfing K. J. (2021). Comparing the effects of a different social partner (social robot vs. human) on children's social referencing in interaction. Front. Educ. 5, 569615. 10.3389/feduc.2020.569615

[B88] Tolksdorf N. F. Mertens U. (2020). “Beyond words,” in International perspectives on digital media and early literacy: The impact of digital devices on learning, language acquisition and social interaction. Editors Rohlfing K. J. Müller-Brauers C. (London: Routledge), 90–102. 10.4324/9780429321399-7

[B89] Tolksdorf N. F. Siebert S. Zorn I. Horwath I. Rohlfing K. J. (2020). Ethical considerations of applying robots in kindergarten settings: Towards an approach from a macroperspective. Int J Soc Robotics 13, 129–140. 10.1007/s12369-020-00622-3

[B90] Tolksdorf N. F. Viertel F. E. Rohlfing K. J. (2021b). Do shy preschoolers interact differently when learning language with a social robot? An analysis of interactional behavior and word learning. Front. Robot. AI 8, 676123. 10.3389/frobt.2021.676123 34136535 PMC8201989

[B91] van den Berghe R. Verhagen J. Oudgenoeg-Paz O. van der Ven S. Leseman P. (2019). Social robots for language learning: A review. Rev. Educ. Res. 89 (2), 259–295. 10.3102/0034654318821286

[B92] Vogt P. de Haas M. de Jong C. Baxter P. Krahmer E. (2017). Child-robot interactions for second language tutoring to preschool children. Front. Hum. Neurosci. 11, 73. 10.3389/fnhum.2017.00073 28303094 PMC5332435

[B93] Vogt P. van den Berghe R. de Haas M. Hoffman L. Kanero J. Mamus E. (2019). “Second language tutoring using social robots: A large-scale study,” in Proceedings oft he 14th ACM/IEEE international conference on human-robot interaction (HRI) (IEEE), 497–505. 10.1109/hri.2019.8673077

[B94] Walker E. Girotto V. Kim Y. Muldner K. (2016). “The effects of physical form and embodied action in a teachable robot for geometry learning,” in 2016 IEEE 16th international conference on advanced learning technologies (ICALT), 381–385. 10.1109/icalt.2016.129

[B95] Wood L. Dautenhahn K. Robins B. Zaraki A. (2017). “Developing child-robot interaction scenarios with a humanoid robot to assist children with autism in developing visual perspective taking skills,” in Proceedings of the 26th IEEE international symposium on robots and human interactive communication, 1–6. 10.1109/roman.2017.8172434

